# Obstetrical Outcomes in Women With a History of Bladder Augmentation

**DOI:** 10.1002/hsr2.70222

**Published:** 2024-11-29

**Authors:** Porela‐Tiihonen Susanna, Jernman Riina, Taskinen Seppo

**Affiliations:** ^1^ Department of Pediatric Surgery Kuopio University Hospital and University of Eastern Finland Kuopio Finland; ^2^ Department of Obstetrics and Gynecology Helsinki University Hospital and University of Helsinki, Helsinki Finland; ^3^ Department of Pediatric Surgery, New Children's Hospital Helsinki University Hospital and University of Helsinki Helsinki Finland

**Keywords:** augmentation cystoplasty, bladder augmentation, cesarean section, delivery, pregnancy

## Abstract

**Purpose:**

To evaluate possible problems during pregnancy or delivery in women with pediatric bladder augmentation.

**Methods:**

Eleven of 59 women, who had undergone bladder augmentation in our pediatric hospital during 1990–2019, had given birth in our hospital district afterwards and their obstetrical records were evaluated.

**Results:**

Median age at first delivery was 32 years (range 26–42). Six patients had myelomeningocele, two had bladder exstrophy and the remainder had VATER association, epispadias or traumatic paraplegia with vesicovaginal fistula. The patients had altogether 18 children (all singletons). Catheterizations were performed through continent stoma in six cases and through urethra in five cases. None of the patients needed an indwelling catheter before delivery. Antibiotic prophylaxis was initially in use during two pregnancies. Symptomatic urinary tract infections (UTIs) developed for five mothers in 11 pregnancies without prophylaxis and prophylaxis was continued after UTI in these cases. Three of the five mothers with UTI were treated with intravenous antibiotics due to pseudomonas infection (three infections) or pyelonephritis (one).

Two patients with myelomeningocele delivered vaginally (one woman three times and one woman once). In the remaining 14 cases a cesarean section (CS) was performed (two urgent and one emergency CS). A urologist was present in seven CSs. Some difficulties accessing the uterus were reported in seven surgeries. There were 10‐term, three late‐preterm and one very preterm delivery. In four cases the information on gestational age was unavailable. Six newborns had respiratory problems, two had severe asphyxia. One newborn had myelomeningocele like her mother.

**Conclusions:**

Risk for UTIs during pregnancy is high in bladder augmentation patients, hence prophylactic antibiotics are justified. A multidisciplinary team should be involved in the planning of delivery. When indicated for obstetrical or urological reasons, an elective cesarean section with a urologist present may be the most rational option for many, although vaginal delivery is possible in selected patients.

## Summary

Women with bladder augmentation should use antibiotic prophylaxis to prevent urinary tract infections during pregnancy. Challenges in cesarean section should be well prepared for with an experienced team including a urologist. Vaginal delivery need not be automatically ruled out.

## 1 Introduction

Augmentation cystoplasty is a procedure performed to increase functional bladder capacity, to protect the upper urinary tract, and to provide continence [1]. The procedure is most often performed for patients with severe neurogenic bladder dysfunction or lack of bladder capacity for other reasons such as bladder exstrophy. Usually, a segment of the intestine is used to enlarge the bladder. In congenital diseases the procedure is often performed during childhood or adolescent age. Sometimes an additional bladder neck procedure is performed to further improve urinary continence, or a continent stoma is constructed to help or enable clean intermittent catheterization (CIC) program [1].

Although bladder augmentation is effective in treating urinary incontinence and protecting the upper urinary tract from hydronephrosis, it predisposes the patient to various problems. Most typical problems are urinary tract infections (UTIs), metabolic acidosis, bladder mucus, and stones and intestinal occlusions [1]. Patients with a background of congenital anomalies such as bladder exstrophy, divergences in sexual development, or complex anorectal malformation may have abnormal or scarred pelvic floor. In female patients, these may affect possible later pregnancy and delivery [2, 3, 4]. Febrile and more severe UTIs, pyelonephritis, may contribute to premature delivery, unlike less severe lower UTIs [5]. Intestinal obstruction is a known but rather rare complication after bladder augmentation and may be more severe a condition during pregnancy [6, 7].

In patients with neurogenic bladder dysfunction, the associated difficulties related to neurological conditions such as possible restrictions in hip mobility and moving and pushing ability may affect the success of vaginal delivery [8]. In patients with abnormal or scarred pelvic floor precludes safe vaginal delivery and cesarean section (CS) is recommended in these cases [9]. Uterine or pelvic organ prolapse is common in patients with bladder exstrophy or myelomeningocele (MMC) and the condition may worsen after pregnancy and delivery [10].

To further elucidate the obstetrical performance of patients with a history of augmentation cystoplasty in a single tertiary hospital, the primary aim of this study was to evaluate the obstetric challenges and outcomes in this infrequent patient group.

## 2 Materials and Methods

Female patients with bladder augmentation were sought from the hospital pediatric urology diagnosis registry of Helsinki University Hospital after permission from the institutional review board (HUS/180/2020). Eleven patients had undergone 18 deliveries. The follow‐up during pregnancy was carried out in the obstetrics department of the same hospital in all except one patient, who was followed up in another hospital district. The data were collected retrospectively from patient records.

The following data were collected: diagnosis, age and type of augmentation procedure, bladder neck surgery or continent stoma procedures; the data concerning the urinary system during pregnancy: the possible findings of bacteriuria and infections, the usage of drugs, reported problems in urinary continence or continuous intermittent catheterization and signs of urinary stasis such as progressive hydronephrosis; information on the pregnancy and delivery: the possible obstetric concerns such as gynecological infections and abnormal antenatal ultrasound findings, and data on the planned mode of delivery and course of the delivery; reported condition of the newborn immediately after birth; postnatal gynecological and urinary tract issues from the follow‐up visit reports after delivery.

In the statistical evaluation, continuous variables are expressed as medians and ranges. Only categorial variables were compared between the groups by using Fisher's exact test for the analyses. The analysis of the data was performed using SPSS Statistics version 27 (IBM Corp. 2020. IBM SPSS Statistics for Windows).

## 3 Results

Bladder augmentation was performed at a median age of 12.2 years (range 6.9–24) during 1988–1997. Six patients had a pure neurogenic disease (myelomeningosele), three had pure structural malformation (two with bladder exstrophy and one with espispadias) and three had both neurogenic and structural disease (one with traumatic paraplegian and vesicovaginal fistula and the other had VATER syndrome with caudal regression and anorectal malformation (Table 1). Ten patients had an ileocystoplasty and one had a coloileocystoplasty (Mainz 1). All patients were on the CIC program and six of them had a continent stoma. Three patients had undergone a bladder neck closure. Nine were ambulatory, two patients used a wheelchair. There were no reported cases of artificial reproduction; 13 pregnancies in eight patients were spontaneous and in five pregnancies the data were not available. Three patients were diagnosed with gestational diabetes.

**Table 1:** Baseline characteristics of patients with augmentation cystoplasty during 1988–1997 and later childbirth during 1997‐2017.

Case	Diagnosis	Operation, age (y)	Continence	Other problems
1	Bladder exstrophy	Coloileocystoplasty and undiversion at 24.0, continent stoma (ureter)	Continent	Metabolic acidosis, bicarbonate medication
2	MMC with HC, shunt, Chiari	Ileocystoplasty at 23.5	Continent	—
3	MMC without HC	Ileocystoplasty at 14.6	Continent	Recurrent urinary tract infections
4	MMC without HC, L1‐L2, Chiari II	Ileocystoplasty and undiversion at 21.6, continent stoma (ureter)	Continent	Recurrent erysipelas, decubitus, left hydronephrosis
5	MMC without HC, L5‐S1	Ileocystoplasty at 11.7	Continent	Urinary stones operated, hypertension
6	MMC, with HC, shunt	Ileocystoplasty at 11.7	Continent	—
7	MMC with HC shunt	Ileocystoplasty at 12.2	Continent	Decubitus ulcerations
8	Paraplegia, traumatic urethral damage	Ileocystoplasty at 14.6, continent stoma (appendix)	Continent	CIC problems
9	Caudal regression. syringomyelia, VATER, vestibular anus	Ileocystoplasty at 6.9	Continent	—
10	Epispady	Ileocystoplasty at 11.9, continent stoma (ureter)	Incontinent	Urethro‐vaginal fistula, urinary stones, MS‐disease hypertension
11	Bladder exstrophy	Ileocystoplasty at 11.1, continent stoma (ureter)	Continent	Urethro‐vaginal fistula, bacteriuria

Abbreviations: CIC, clean intermittent catheterization; HC, hydrocephalus; MMC, myelomeningocele; MS, Multiple sclerosis; PSARP, posterior sagittal anorectoplasty; VATER, VATER association; a multiple anomaly disorder.

A variable degree of urinary incontinence was reported in one patient (9%) before pregnancy and in six (55%) patients during pregnancy (*p* = 0.064). Incontinence occurred either through urethra or through stoma. Five patients (45%) reported difficulties to perform CIC during pregnancy. Two of them had a continent urinary stoma and three were catheterized via urethra. None of the patients needed an indwelling catheter before delivery or CS. Other reported problems were the feeling of incomplete bladder emptying and need to perform CIC more frequently.

Information of possible UTI and bacteriuria was available in 16 of 18 pregnancies. Eleven of 16 cases had symptomatic UTIs (one with pyelonephritis) and two cases had asymptomatic bacteriuria. Pseudomonas infection requiring intravenous antibiotic therapy occurred in three pregnancies. Two patients were on antibiotic prophylaxis before any infection. After UTI or detection of asymptomatic bacteriuria antibiotic prophylaxis was initiated in all 13 pregnancies. However, in nine of them repeated UTI:s from one to five times. In two pregnancies there were neither antibiotic prophylaxis, bacteriuria nor infections. There were no cases of pre‐eclampsia, but three patients required medication for hypertension during pregnancy.

The patients delivered during the years 1997–2017 (Table 2). Three patients delivered three times, one delivered twice and the remaining seven patients each delivered once. Of the 18 deliveries, 14 (78%) were CSs (eight elective, two urgent, one emergency, and three of unknown CS) and four (22%) were spontaneous vaginal deliveries. The decision‐to‐incision interval in emergency CS was 10 min, and 30 min or more in urgent CS, based on the obstetrician's evaluation. One vaginally planned delivery was converted to emergency CS due to signs of imminent fetal asphyxia.

**Table 2:** The pregnancy outcomes (mode of delivery and information on the newborns) of patients with augmentation cystoplasty during 1988–1997 and childbirth during 1997–2017.

Case	Diagnosis of mother Type of bladder dysfunction	Mode of delivery	Gestational age at delivery, gender and birthweight	Health status of the newborn	Other notes
1	Bladder exstrophy Non‐neurogenic	Elective CS	39 + 2 girl, 2531 g	Healthy, hip instability	—
2	MMC with HC, Chiari Neurogenic	CS (N/A)	N/A,	Healthy	Uterine prolapse operated after 2nd pregnancy
			girl		
		CS (N/A)	N/A	N/A	
3	MMC Neurogenic	Vaginal delivery	38 + 0	MMC	Uterine prolapse after 1st pregnancy
			girl, 2700 g	Healthy	
		Vaginal delivery	37 + 4	Healthy	
			girl, 2954 g		
		Vaginal delivery	39 + 4		
			girl, 3096 g		
4	MMC L1‐L2, Chiari II Neurogenic	CS (N/A)	N/A	N/A	—
5	MMC L5‐S1 Neurogenic	Urgent section (imminent asphyxia)	26 + 2	Asphyxia, prematurity	Umbilical cord entanglement
			girl, 670 g		
6	MMC with HC	Emergency CS (imminent asphyxia)	36 + 2	Severe asphyxia	Trial of labor in 1st pregnancy
			boy, 2540 g		
	Neurogenic	Elective CS		Healthy	
		Urgent CS (spontaneous rupture of membrabes)	39 + 1	Healthy	
			boy, 2945 g		
			38 + 2		
			girl, 3090 g		
7	MMC with HC	Vaginal delivery	N/A	N/A	Rapid unplanned home delivery, uterine prolapse after pregnancy
	Neurogenic				
8	Traumatic paraplegia, urethral damage	Elective CS	37 + 0	Healthy	—
			boy, 3120 g		
	Neurogenic	Elective CS	37 + 3	Healthy	
			boy, 3300 g		
		Elective CS	36 + 6	Healthy	
			girl, 2415 g		
9	Caudal regression, VATER	Elective CS	38 + 2	Healthy	Bladder lesion sutured in CS
	Neurogenic		girl, 3095 g		
10	Epispady	Elective CS	35 + 4	Healthy	—
	Non‐neurogenic		boy, 2340 g		
11	Bladder exstrophy	Elective CS	38 + 3	Healthy	—
	Non‐neurogenic		boy, 3018 g		

N/A: Data not available. Abbreviations: CS, cesarean section; HC, hydrocephalus; MMC, myelomeningocele; VATER, VATER association; a multiple anomaly disorder.

Of the 14 Cesarean sections, the decision to perform a CS was due to anatomic condition in 10 cases, due to obstetric condition (imminent asphyxia) in two cases and unknown in two cases. During CSs, some difficulties entering the uterus were reported in seven out of 14 (50%) surgeries. In the remaining cases, the augmented bladder and pedicle had slid laterally making access to the uterus easy. In six of the more difficult cases, the augmented bladder had to be surgically prepared off the uterus. In one such case, this resulted in a small, one‐centimeter tear in the urinary bladder which was sutured and no other interventions were needed. In six cases the access to the uterus had become slightly difficult because of scarring and adhesions. The mean intraoperative blood loss was 716 mL (range 200–1410 mL). A urologist was present in seven (50%) surgeries.

Two patients with MMC delivered vaginally, one woman three times and the other had an unplanned rapid home delivery. The vaginal deliveries were spontaneous. Oxytocin was used for enhancing contractions in two deliveries. The second stages of labor in these deliveries were uneventful. There were no complications such as severe perineal tears. Minor perineal tears that required one to four stitches occurred in two deliveries.

The gestational age of the newborn was available in 15 cases. Eleven of the newborns were full‐term (≥ 37 gestational weeks), three were late preterm (34 + 0‐36 + 6), and one was extremely preterm (< 28 + 0). Six newborns required respiratory assistance immediately after birth. Two of them were born by urgent or emergency CS due to imminent asphyxia, including the extremely preterm neonate. One of the newborns had MMC like her mother despite folic acid supplementation. The outcome of the newborns is reported in Table 2.

## 4 Discussion

We report the obstetric outcomes of women with a history of bladder augmentation treated in the largest tertiary hospital in Finland. Six mothers had pure neurogenic disease, three had pure structural malformation and three had cobined neurogenic and structural disease. Almost all pregnancies in this series were complicated by UTIs and antibiotic prophylaxis was used thereafter during the rest of the pregnancy. Despite the high prevalence of UTIs, most (61%) of the newborns were born at term. None of the mothers developed pre‐eclampsia or any severe pregnancy complications, but three required hypertension medication. Only two mothers with neurogenic disease gave birth vaginally, one of whom had an unplanned home delivery, and both mothers experienced later problems with uterine prolapse. In one additional case an attempt to vaginal delivery ended in emergency CS. During CS, the urinary bladder or its vascular pedicle was attached high on the anterior uterine wall in six of 14 cases making the operation more challenging, but there were no serious adverse events. Most of the newborns were in good health, although six required respiratory assistance immediately after birth.

The prevalence of asymptomatic bacteriuria is close to 1%–2% in pregnant women and about 30% of them develop symptomatic UTIs if left untreated, compared to 1.8% of nonbacteriuric controls [11]. The occurrence of bacteriuria after bladder augmentation varied from 45% to 84% in one study [12] and the incidence of symptomatic infections during the first 10 postoperative years is reported as high as 28%–51% in three large series even without pregnancy [13, 14, 15]. Accordingly, the incidence of serious infections during pregnancy is markedly higher (32%–100%) after urinary tract surgery [2, 3, 4, 16, 17, 18]. Similarly, almost all of our mothers had problems with UTIs and antibiotics were used in all except two pregnancies.

Infections may contribute to premature labor [5]. Treatment of asymptomatic bacteriuria is commonly recommended to avoid pyelonephritis and premature labor in all pregnant women [5]. Despite the risk for severe UTIs during pregnancy seems to be high after bladder augmentation, only 12% of the patients had prophylactic antibiotic in a recent review [16].

We have not found guidelines on antibiotic prophylaxis should be routine in patients with bladder augmentation, and the role of prophylaxis is controversial [19, 20]. Our results support the active treatment of even asymptomatic bacteriuria in bladder augmentation patients during pregnancy. In addition to increased risk of premature delivery, severe, although rare complications of UTI include pyelonephritis and urosepsis [5].

However, despite the several UTIs in our series, only four patients delivered preterm, one extremely preterm and three late‐preterm; the latter group of neonates has higher morbidity compared to term neonates, but generally manage well [21]. In these four pregnancies, there were no cases of pyelonephritis, but two had multiple cystitis. Two of these preterm deliveries were urgent or emergency CSs performed due to imminent asphyxia, and two were CS deliveries performed late‐preterm due to premature cervical ripening.

Other problems discovered in our series were difficulties in emptying the bladder with a catheter via stoma or urethra (CIC procedure), but none of the patients needed a Foley catheter even at the end of pregnancy before delivery. Similar difficulties are described previously in up to 100% of pregnant women with previous urinary tract reconstruction [17]. In the study of Hensle et al. [17] all 11 pregnant women with urinary tract reconstruction had difficulties in CIC and four needed an indwelling catheter. More difficulties are described in right lower quadrant stomas than with umbilical stomas [2, 16]. In addition, ureteral obstruction is possible during pregnancy. In the review of Bey et al. (2021) [16], urinary diversion (nephrostomy or ureteral stenting) was required during pregnancy in 11% of the women included. However, none of our patients needed ureteral stenting.

The mode of delivery needs to be planned carefully in women with lower urinary tract reconstructions. The patient's basic diagnosis, anatomy of the pelvis and hips, previous surgery, and general condition must be taken into consideration. High spinal cord injury above the T6 level, rigid hips and abnormal pelvis anatomy may prevent or complicate vaginal delivery [16]. In severe structural anomalies such as bladder exstrophy, complexed anorectal malformation and cloacal exstrophy with previous complex pelvic surgery, vaginal delivery is not recommended [2, 22]. Overall, previous studies have reported CS performed in 48%–68% of the patients with MMC or other major neurogenic diseases [20, 23, 24, 25]. Shepard et al. (2019) reported 9516 deliveries in patients with spina bifida. Cesarean section was more common in patients with spina bifida compared to other women (53% vs. 32%), but there was no significantly increased complication risk associated with vaginal delivery compared to women without spina bifida [25]. After major lower urinary tract reconstructions, CS is more common than vaginal delivery [2, 17, 18]. Also in our series, only three of 11 mothers attempted vaginal delivery. Two of them indeed succeeded but the third delivery ended to emergency CS. During CS, there were some difficulties to enter the uterus either because of the deviant location of the bladder in the front of uterus or because of adhesions in half of our cases. Obstetricians may thus have felt the presence of a urologist reassuring. Patients who have undergone an augmentation cystoplasty surgery in childhood or juvenile years are an uncommon patient group that obstetricians rarely meet. A urologist can be of aid in evaluating the anomalous anatomical circumstances and possible neurogenic issues during delivery and prevent procedure‐related complications to the urogenital tract.

In one of the CS surgeries occurred a small bladder wall perforation. Previously concerns have been expressed relating to direct injury to the neobladder or its blood supply and difficulties or delays in reaching the uterus [26, 27]. We agree with the previous recommendation that it is advisable to insert a catheter to continent stoma during CS to prevent its injury [16].

Among our patients, any degree of urinary incontinence increased from 9% before pregnancy to 55% during pregnancy. Others have described similar increased incontinence rates [17]. On the other hand, the findings of reduced cystometric capacity of the bladder and increased urinary frequency are associated to pregnancy also in healthy women [28]. Postpartum incontinence has been previously reported in bladder augmentation patients, especially after vaginal delivery [27]. However, in a recent review, only 4% experienced incontinence more than 3 months after delivery and the mode of delivery was not associated with incontinence [16]. Similarly, in all our patients the incontinence subsided after delivery and 10 of 11 patients reported good continence at their latest urological control visit.

Four of our patients delivered preterm (one extremely preterm at 26 gestational weeks) and two newborns had asphyxia, both were delivered by urgent or emergency CS due to imminent asphyxia. Also, there was one rapid unplanned home delivery. Patients may not always feel contractions, which can sometimes provoke fetal stress and lead to hypoxia. Overall, in most cases the outcome of the newborns was good. In general, preconceptual and antenatal folic acid supplementation is recommended to prevent neural tube defects (NTD) and for women at high risk such as MMC patients, the dose is also higher [29, 30]. One of our patients who had MMC also delivered a child with MMC, despite having used preconceptual folic acid (the dose was unknown). Furthermore, there were 1–2 suspected earlier MMC cases in her family. Overall, the use of folic acid was documented in only two of our patients (deliveries in 2005, 2008, and 2010), thus it is unclear how many women actually used folic acid or were aware of the recommendation.

Our study has some limitations. The number of pregnancies was small [18] during 20 years of follow‐up. The study design was a retrospective analysis, making the collecting of all details challenging at times. Nevertheless, all significant findings and problems were reported adequately and obstetrical information was mostly well documented in the patient files.

## 5 Conclusions

In conclusion, the recommendation to use prophylactic antibiotics seems appropriate in bladder augmentation patients to prevent UTIs during pregnancy. Even though most of our patients underwent CS, in patients with neurogenic diagnoses and previous pelvic surgery, vaginal delivery need not to be automatically ruled out. Due to earlier operations and anomalous anatomy, surgical challenges during CS should be anticipated and be well prepared for with an experienced team, ideally including a urologist.

Social awareness of reproductive equality has increased during the last years, and pregnancies are possible also for women with severe disabilities. A multidisciplinary team is essential in planning the follow‐up of the pregnancy and delivery.

## Author Contributions

**Susanna Porela‐Tiihonen:** conceptualization (supporting); investigation (equal); formal analysis (equal); writing – original draft (lead); writing – review and editing (equal). **Riina Jernman:** investigation (equal); formal analysis (equal), writing – original draft (supporting); writing – review and editing (equal). **Seppo Taskinen:** project administration; conceptualization (lead); investigation (equal); formal analysis (equal); writing – original draft (supporting); writing – review and editing (equal).

## Disclosure

The lead author Porela‐Tiihonen Susanna affirms that this manuscript is an honest, accurate, and transparent account of the study being reported; that no important aspects of the study have been omitted; and that any discrepancies from the study as planned (and, if relevant, registered) have been explained.

## Ethics Statement

The study was conducted in accordance to the declaration of Helsinki. The ethical approval for this study was obtained from the Helsinki University Hospital institutional review board (HUS/180/2020).

## Conflicts of Interest

The authors received no financial support for the research, authorship, and/or publication of this article. There are no conflicting interests in any author (SP‐T, RJ, ST).

## Data Availability

The data that support the findings of this study are available on request from the corresponding author. The data are not publicly available due to privacy or ethical restrictions.
